# Measuring Veterinarian Professions’ Readiness for Interprofessional Learning in a Pre- and Post-Intervention Study

**DOI:** 10.3390/ani14020229

**Published:** 2024-01-11

**Authors:** Sylva Agnete Charlotte Heise, Andrea Tipold, Karl Rohn, Christin Kleinsorgen

**Affiliations:** 1 E-Learning-Consulting, Center for E-Learning, Didactics and Educational Research, University of Veterinary Medicine Hannover, 30559 Hannover, Germany; 2 Clinic for Small Animals, University of Veterinary Medicine Hannover, 30559 Hannover, Germany; andrea.tipold@tiho-hannover.de; 3 Institute of Biometrics, Epidemiology and Information Processing, University of Veterinary Medicine Hannover, 30559 Hannover, Germany

**Keywords:** interprofessional, blended learning, teamwork, RIPLS, communication

## Abstract

**Simple Summary:**

Interprofessional learning is increasingly important across all health care sectors, including veterinary medicine. Communication with animal owners and within the team is considered a core competency. As communication courses have been offered mostly on a voluntary basis, we conducted an interprofessional learning course for veterinary practice professions in this study with the aim of integrating such a course in the curriculum in the long term. We measured the readiness using the Readiness for Interprofessional Learning Scale before and after the course. While few significant changes emerged in the pre- and post-test comparison, more notable differences were observed in the evaluation of the 19 statements among different professions.

**Abstract:**

The integration of interprofessional collaboration is becoming increasingly crucial in veterinary care settings, emphasising the need for interprofessional education (IPE) in veterinary programmes. This study explores the readiness for interprofessional learning among German veterinary students, apprentices and related occupations before and after an interprofessional communication course. It assesses the impact of this course on the participants’ attitudes using the Readiness for Interprofessional Learning Scale (RIPLS). The course, offered in two iterations, combined asynchronous online modules, live seminars and practical training elements. The RIPLS was administered before and after the course to gauge attitude shifts towards interprofessional learning. Statistical analyses, including McNemar, Cohen’s Kappa and exact Fisher tests, were employed to compare pre- and post-test responses. Despite challenges in participant linking, significant findings emerged between the student and apprentice groups in specific areas of the RIPLS, notably in the “Professional Identity” subscale post-course. However, correlations between face-to-face contact and RIPLS ratings were not observed, suggesting a need for more integrated interprofessional learning experiences. While some limitations in sample size and profession distribution hinder generalisability, this study indicates a high receptiveness to interprofessional learning in veterinary education, emphasising the potential for attitude changes with more interactive participation and programme adjustments.

## 1. Introduction

Patient care in veterinary practices is increasingly handled by persons from different professional groups who must work together to effectively care for patients [1] (Kinnison et al., 2014). In the German veterinary medicine degree study programme, the first intensive interprofessional contact typically occurs during the final year of study, the practical year. To facilitate the transition to professional life, interprofessional events during undergraduate education or apprenticeships could be beneficial.

Interprofessional teamwork is gaining importance in all health care sectors. Studies indicate that treatment success relies not only on expertise but also on effective collaboration [2] (Antoni, 2010). While communication and, in some cases, explicit interprofessional communication have already been integrated into learning objectives in many health care fields, communication training in the field of veterinary medicine is not embedded [3,4], but often conducted on a voluntary basis at the five veterinary institutions in Germany, typically in the form of elective courses with a focus on patient communication [5]. Previous needs assessments have indicated that adjustments are required in veterinary medicine, including the need for communication training [6]. Furthermore, there have been suggestions at a high level for curriculum adjustments in order to integrate interprofessional approaches [7]. In the training catalogue for veterinary assistants’ communication with owners is also emphasised, but no interprofessional communication is mentioned [8].

Although the veterinary team may comprise various professions such as practice managers, physiotherapists, nutritionists, etc., this study specifically focuses on veterinary students, doctoral students, veterinary assistants and trainees, animal keepers and trainees and biology laboratory technicians. The occupations of veterinary assistants, animal keepers and biology laboratory technicians are nationally recognised and require certification in Germany, involving a three-year training period. Veterinary assistants aid veterinarians in animal examinations, treatments and care and provide guidance to the animal owners. They also handle organisational and administrative tasks. Animal keepers can also find employment in veterinary practices where they are responsible for animal care and provide support for procedures and treatments. Both professions frequently interact with clients as part of their daily professional responsibilities [1]. Biology laboratory technicians typically have less contact with the clients, although they sometimes communicate with owners about testing results. Nonetheless, they also play a crucial role in the diagnostic and analytical aspects of animal health. They need to work hand-in-hand with the other professions to collect samples, prepare them for diagnostic testing, interpret the results and prepare the reports. Veterinary medicine study courses in Germany take eleven terms (resulting in 5 ½ years), with most practical internships in veterinary practice occurring during the ninth and 10th term [9].

Hattie et al. [10] define learning interventions as extracurricular activities intentionally designed to improve academic performance. These activities go beyond mere content learning and also aim to instill learning-related skills such as self-efficacy and study techniques. Similarly, an interprofessional learning intervention includes learning outcomes beyond cognitive competences and addresses affective learning outcomes, involving attitudes, motivation and values [11].

The Readiness for Interprofessional Learning Scale (RIPLS) is a validated measurement instrument originally developed for human medicine to assess the readiness for interprofessional learning. It consists of 19 statements divided into three sections: (1) teamwork and collaboration, (2) professional identity and (3) roles and responsibilities [12]. It was initially developed for use with students in health care disciplines but has also been validated for graduates [13]. The originally English questionnaire has been translated into German by various institutions, including Heidelberg University and Martin Luther University Halle-Wittenberg [14]. Both versions were methodologically reviewed and compared, although not all sections yielded acceptable reliability values. The use of a German translation in the context of a bachelor’s programme in “Interprofessional Health Care” showed limitations, particularly in the third section “Roles & Responsibilities” [15]. Nevertheless, the RIPLS and its translations provide a valuable starting point for interprofessional needs assessment. In human medicine, it has already been observed that professional identification has various effects on attitudes towards interprofessional learning [16]. Nursing students showed a more positive impact than medical students, and interprofessional contact with different professional groups did not significantly influence the results. The observations are also supported by social identity theory, which illustrates that social position in a social structure significantly contributes to a sense of belonging [17]. Members of groups with lower status tend to place more importance on their group identity compared to those with higher status if it can increase the value of their own group.

The RIPLS questionnaire has already been applied comparatively to medical doctors and veterinarians, with results indicating that the need for teamwork is higher in veterinary medicine [18]. In 2021, a high relevance of communication skills and a strong interest in teaching these skills as part of an interprofessional education were identified in veterinary medicine [19]. Based on these findings, an interprofessional learning intervention was developed and implemented at the University of Veterinary Medicine Hannover (TiHo Hannover), Germany. This study assesses the impact of an interprofessional learning course using the RIPLS to quantitatively measure readiness for interprofessional learning. The research question to be examined is how evaluation of the relevance of interprofessional learning, using the RIPLS, changes through the interprofessional communication intervention?

## 2. Materials and Methods

**Figure 1:**
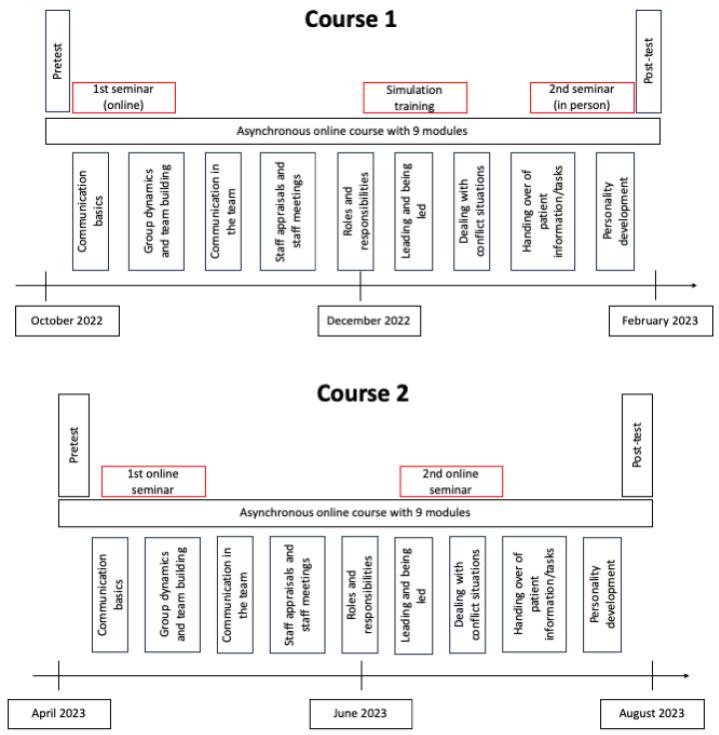
The blended learning course design for the winter term 2022/2023 (Course 1) and for the summer term 2023 (Course 2); differences in the design are highlighted in red.

At the TiHo Hannover, an interprofessional learning intervention was conducted as an elective course in the winter term of 2022/2023. The course was made available for veterinary students and veterinary assistant trainees from the Veterinary University Hannover. It was designed as a blended learning course consisting of an asynchronous online course comprising nine modules on the learning management system Moodle. This course design should facilitate participation from individuals across different professional groups and accommodate their differing daily schedules accordingly. While most theoretical content was delivered within self-learning phases, interaction between participants and course directors was facilitated during online and in-person seminars. Within the modules, different theoretical content was delivered, supplemented with video materials and interactive tasks. The modules’ topics were developed in alignment with the core competencies outlined by the WHO [7] and the recommendations provided by the Interprofessional Education Collaborative [20]. The IPEC core competencies were used as a basis, as they have been developed, revised and already incorporated into curricula and accreditation standards for several years. The processing time for each module was designed to be around two hours, whereas the processing period for participation ranged from one to three weeks depending on its scope. Upon completion of each module, participants were required to complete tasks to validate successful engagement. These tasks encompassed quizzes, interactive videos, audio recordings or text composition. As part of the course preparation, thematically relevant topics were collected and discussed by course facilitators. Videos were then produced as either best practice or worst case examples, with professional actors highlighting typical situations and conflicts encountered in the veterinary practice. Additionally, one synchronous online seminar on the topic of roles and responsibilities, prejudices and conflict potentials, one in-person seminar led by a communication coach on resilience and stress management and a simulation training with professional actors to apply the theoretically learned content were included (see Figure 1, Course 1). To assess the demand for interprofessional learning, the RIPLS was administered before and after the course using the survey tool LimeSurvey^®^ (LimeSurvey GmbH, Version 3.23.1, Hamburg, Germany) in accordance with the translation by the Martin Luther University Halle-Wittenberg [14]. Instead of the four-point scale version used by the Martin Luther University Halle-Wittenberg, a five-point Likert scale, as in the version implemented by Heidelberg University, was used [14,21]. Furthermore, a comment was added that under the term “health care students”, participants should include veterinarians, vet nurses, vet technicians and others. Moreover, under the term “doctor”, they may also consider veterinarians. The items of the RIPLS are numbered from 1 to 19 in the following and belong to three subscales (Table 1). The RIPLS questionnaire was part of the pre- and post-test, which also included general questions about personal information and previous experiences, a knowledge test and self-assessment of their own communication skills. Additionally, expectations for the course were surveyed in advance, followed by a comprehensive evaluation in the post-test.

In the summer of 2023, the course was offered again as an elective course, but without face-to-face events due to resource limitations (see Figure 1, Course 2). This meant that the simulation training was omitted and the second seminar was held online instead of in person. However, it was made available to all trainees and fully trained professionals in various veterinary practice occupations at the TiHo Hannover as well as to students and doctoral candidates. These changes in the course design may influence this study’s results. Hereafter, we will group all of the vocational training professions that participated under “apprentices” (veterinary assistants and trainees, animal keepers and trainees, laboratory technicians and trainees) and all of the veterinary students and doctoral candidates under “students”.

The surveys carried out were subject to general data protection regulations. Written consent to participate in the study was obtained from all participants before answering the questionnaires. Furthermore, an individualised code was self-generated by each participant following an instruction so that an anonymised, paired sample could be collected. Participation was voluntary and anonymous. Apart from the unprocessed IP address, no data were collected that would have allowed conclusions to be drawn about the identity of the respondents. Each question could be skipped or answered with “no answer”.

Pre- and post-test data were exported from LimeSurvey^®^ (Version 3.23.1+200825) as Microsoft Excel (Version 16.80 (23121017)) spreadsheets, and SAS 9.7M7 with Enterprise Guide 7.1 (SAS Institute Inc., Cary, NC, USA) was used for the analysis of the collected data.

To calculate intrarater reliability, the McNemar and Bowker tests and Cohen’s kappa with PaBaK (prevalence and adjustment) were used for statistical analysis. Fisher’s exact test was used to calculate the group differences. The statistical test McNemar was used for paired data in 2 × 2 tables (Bowker test for n × n tables, where n is greater than 2) and it was applied to examine situations where the same subjects or elements are measured under two different conditions, e.g., at two different time points. In our case, it was applied to examine the symmetry of responses given in the pre- and post-test and to identify any deviations. The probability of error for the McNemar test alpha was set to 5%, i.e., the assumption of the symmetrical bias is rejected with a *p*-value < 0.05.

To assess the agreement of the responses between the pretest and the post-test, the concordance index kappa was calculated. In our case, the prevalence- and bias-adjusted kappa coefficient (PaBaK) was calculated because it is more suitable for the asymmetric distributions between the cell frequencies than the simple kappa coefficient. For the interpretation of the concordance index kappa, we used the table by Landis and Koch [22] as a reference point.

- <0 no agreement.
- 0–0.20 slight agreement.
- 0.21–0.40 fair agreement.
- 0.41–0.60 moderate agreement.
- 0.61–0.80 substantial agreement.
- 0.81–1.0 perfect agreement.

Significant differences in the responses to the RIPLS were determined using Fisher’s exact test. This test was used because the probability of the observed cell contents of the contingency table are often less than five and therefore the chi-square test may become inaccurate. The probability of error alpha for Fisher’s exact test was set to 5%, i.e., the assumption of equality of both professions is rejected at a *p*-value < 0.05.

Due to focus of the research question of this study, the results of the knowledge test, self-assessment and course evaluation were not included; thus, in the following, only results regarding the assessments of the Readiness for Interprofessional Learning Scale are illustrated.

## 3. Results

### 3.1. Descriptive Analysis

In the first course, in the winter term 2022/2023, a total of 18 veterinary students and 13 prospective veterinary assistants were enrolled. In total, 22 questionnaires could be paired by individual codes. In the second course, in the summer term 2023, a total of 81 participants registered for the course, including 63 students, 2 doctoral candidates and 16 who had completed their apprenticeship training (1 of them had yet to complete their training) in veterinary practice occupations. For the evaluation, 59 questionnaires from the pretest and 54 from the post-test were analysable. In this course, a total of 38 paired samples were identified by individual codes. In the following, we will focus on the paired samples of both courses. The demographic data of the course participants with paired samples identified in pre- and post-tests from both courses are shown in Table 2. The majority of the participants had never been part of a communication training before (n = 44, 73.33%), but the majority had already worked in a team (n = 55, 91.67%) and a similar amount had already participated in team activities (e.g., sports, music) (n = 54, 90.00%).

Descriptive statistics of the overall ratings of the RIPLS items in pre- and post-test during both courses are illustrated in Table 1. The statement that received the highest median approval in the pretest was the same as in the post-test, namely, item 2: “Patients ultimately benefit when health professionals work together to solve patients’ health professions.” Item 10—“I do not want to waste my time learning together with members of other care professions”—earned the least approval in both tests.

**Figure 2:**
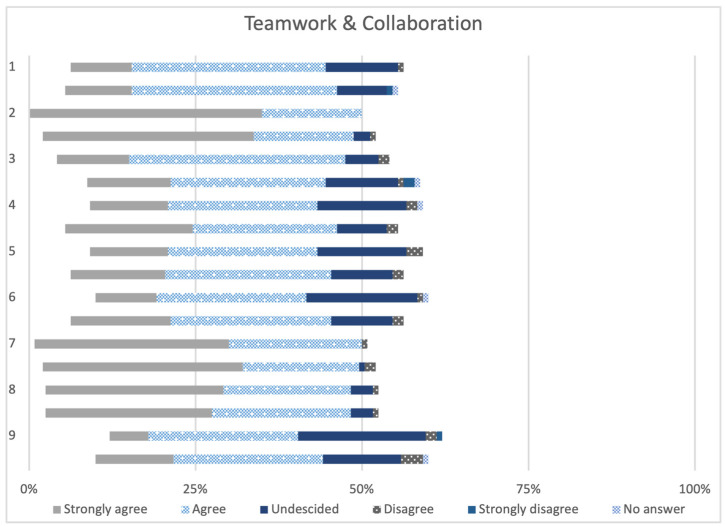
Answers to items 1–9 of the subscale “Teamwork & collaboration” of the pretests and post-tests of both courses (paired samples, n = 60). The items 1–9 of the subscale “Teamwork & collaboration” are shown in Table 1.

In Figure 2, the first nine items of the RIPLS are presented, covering the subscale “Teamwork and collaboration”. For each question, the pretests and post-tests of both courses were combined and compared for all paired samples. This subscale consists of positive statements regarding interprofessional learning, and statements were predominantly rated in agreement by the participants.

**Figure 3:**
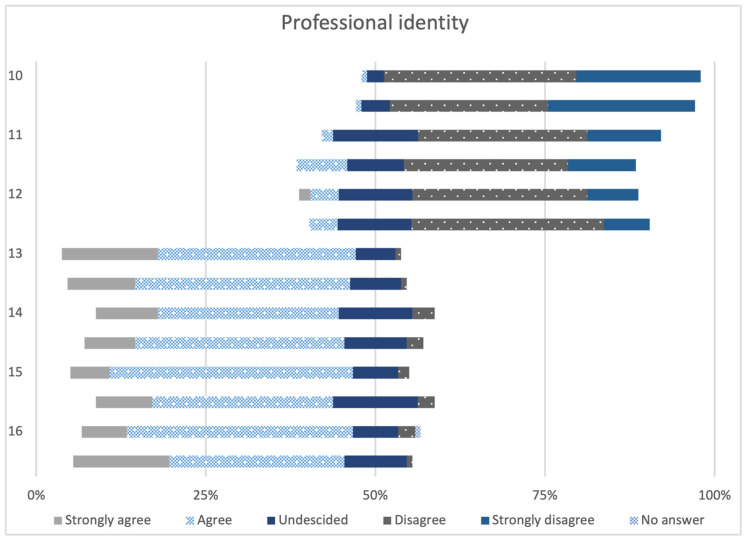
Answers to items 10–16 of the subscale “Professional identity” of the pretests and post-tests of both courses (paired samples, n = 60). The items 10–16 of the subscale “Professional identity” are shown in Table 1.

In Figure 3, items 10 to 16 of the RIPLS are presented which constitute the subscale of “Professional identity”. For each statement, the pretests and post-tests from both courses were combined and compared for all paired samples. Items 10 to 12 consist of negatively formulated statements about interprofessional communication. These mainly received little to no agreement. Items 13 to 16 are positive statements, which were mainly rated positive by the participants.

**Figure 4:**
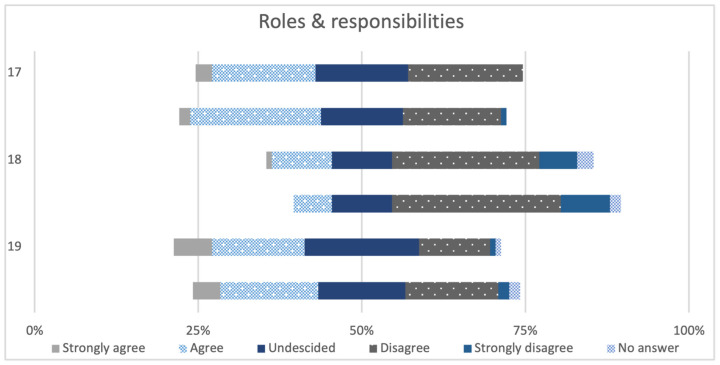
Answers to items 17–19 of the subscale Roles and responsibilities of the pretests and post-tests of both courses together (paired samples, n = 60). The items 17–19 of the subscale “Roles & responsibilities” are shown in Table 1.

The last three items of the RIPLS are presented in Figure 4. These constitute the subscale “Roles and responsibilities”. Again, for each statement the pretests and post-tests from both courses were combined and compared for all paired samples. These statements were answered in a more varied manner. Concerning the item “The function of nurses and therapists is mainly to provide support for doctors”, in the pretest, about half of the apprentices (53.85%, n = 7) agreed with the statement, in contrast to the percentage of students who chose “Strongly agree” and “Agree” less often (“Strongly agree”: 6.38%, n = 3; “Agree”: 25.53%, n = 12). In the post-test, there were similar differences between the two professions; the apprentices rated the statement more positively than the students. The item “I’m not sure what my professional role will be” was answered with more disagreement in the post-test than in the pretest by both professions. The last statement “I have to acquire much more knowledge and skills than other health care students“ was also rated with more agreement in the pretest than in the post-test.

### 3.2. Interference Statistical Analysis

In the total of all pre- and post-tests, mainly fair to moderate agreement between the answers of the pre- and the post-test of both professions was apparent using the Cohen’s (PaBaK) kappa test. Only item 12 had a slight agreement. In the analysis of the students as a separate group, the findings showed mainly fair to moderate agreement. Three items (items 6, 12 and 19) had a slight agreement and item 8 had a substantial agreement. On the other hand, the results of the apprentices mainly showed a slight to moderate agreement. Only item 12 showed no agreement at all. All results of the McNemar test are presented in Supplementary Table S1.

The statement “Shared learning will help me to think positively about other professionals” was answered highly significantly (*p* = 0.0098) differently by the two professions in the pretest. In the post-test, the statement “Shared learning will help to clarify the nature of patient problems” was rated also significantly (*p* = 0.0366) differently by the professions.

Furthermore, there were significant differences when looking at the data from both courses together: in the post-test, almost three different statements of the RIPLS were answered significantly differently by the professions. The statement “It is not necessary for undergraduate health care students to learn together” scored a highly significant *p* = 0.0089 in Fisher’s test. “Shared learning with other health care students will help me to communicate better with patients and other professionals” was rated significantly differently (*p* = 0.0133) and “Shared learning will help to clarify the nature of patient problems” received a value of *p* = 0.0375 and showed also a significant difference. All other exact Fisher tests had a *p* = >0.05 and showed no significant differences in the responses of students and apprentices (Supplementary Table S2).

## 4. Discussion

In this study, we explored the readiness for interprofessional learning of German veterinary students and apprentices (including those who had already completed apprenticeship training). We compared the attitudes before and after an interprofessional communication course to find out whether a common event effects any change in assessment of the 19 statements of the RIPLS. Despite challenges in participant linking, significant findings emerged between the student and apprentice groups in specific areas of the RIPLS, notably in the “Professional Identity” subscale post-course. Regarding the research question, there appears to be no overall improvement in readiness for interprofessional learning following the 4-month course. Only in item 12 is there a lack of agreement between the pre- and post-tests. Notably, there are several significant differences in the assessment of the statements that differ between students and apprentices.

Both courses taken together resulted in a total of 88 evaluable pretests, 78 post-tests and 60 paired samples linked by an individual code. Only the linked samples were included in the analysis; the remaining questionnaires could not be included due to missing links caused by incorrect codes. These problems were also reported in a previous study on learning interventions [23]. The participants received instructions on how to create an individual, anonymous code based on their personal data. While the instructions for the pretest and post-tests were identical, different codes were generated in some cases. These observations should be taken into account in the future, and a simpler instruction could be a possible solution to avoid unassignable datasets. The validated RIPLS, created in 1999 [12], has filled an empirical gap and continues to be a tool for measuring the attitudes of students in health professions towards interprofessional learning. Other questionnaires like the CSAS (Communication Skills Attitude Scale) also exist to measure positive and negative attitudes towards learning communication skills [24] but without the focus on interprofessional learning. However, there are indications that the questionnaire is particularly unsuitable for before-and-after studies, as it inadequately reflects changes in attitudes [25]. This could be based on the assumption that the elements which are relevant for the stable factor values are more strongly correlated with each other than initially assumed. Based on these indications, it must be determined whether a measuring instrument for this specific case does not yet exist. Other studies describe low values of internal consistency in the Cronbach’s alpha method in the subscales, although the values overall show higher reliability [15]. Furthermore, studies indicate that the psychometric integrity of various measurement instruments for interprofessional education is limited [26]. The observation that increased interprofessional contact leads to decreased ratings of the RIPLS items has already been made in Brazil [27]. This is an interesting result which could indicate that the results could be lower after an interprofessional event. However, there is also evidence that interactive communication training promotes learning success [28], which makes the assumption plausible that the attitudes change through such training. In 2021, there was already a needs assessment towards interprofessional training, in which participants of all five veterinarian institutions underlined their wish to participate in such a training [6]. That finding is supported by our result in item five of the RIPLS, namely, “Communication skills should be learned with other health care students”, which was mainly positive in the pre- and post-tests. In addition, we have many different professions working together in veterinarian practice [1], which is why it is important to pay more attention to interprofessional communication. The negative formulated items 10 to 12 mainly received little to no agreement, which illustrates that the participants read the statements attentively and were not distracted due to the length of the whole questionnaire.

In our study design, we compared two different courses with varying face-to-face contact between the professions to check for a correlation between the two different types of courses and the rating of the RIPLS. It is plausible that there was no correlation between contact or shared learning of different professional groups and the assessment of the items, as in our study design, a clear increase in ratings could not be observed. It is noteworthy that during the planning phase of the first course in 2022, finding dates and times during which both participating professions were available was challenging. Providing one live online seminar during the second course was similarly problematic. It became clear that although efforts were made to create joint and interprofessional learning interventions, the current timetables and schedules of different professions do not easily allow for this. In addition, a comparison was made to determine whether different professions exhibited significantly different readiness for interprofessional learning before and after the course. In this context, differences in readiness between medical students and nursing students have already observed [29]. This observation might be applicable to veterinary medicine students and prospective veterinary medical assistants as well. In our datasets, we could also find some significant differences in the rating of some of the RIPLS items between students and the apprentices in specific questions, especially in the overall post-tests under the subscale “Professional identity”. In other countries, significant differences in the response to the various subscales were observed among the different health care groups [30].

The translations of the English language RIPL scale vary, which may lead to a conceptual imbalance that affects its utility, whereas the translation from English into other languages has proved to be reliable in initial tests [31]. Nonetheless translations could lead to variations in the meaning of certain words or phrases, as different languages might not perfectly align. Changes in wording or structure could impact the scale’s psychometric properties, such as internal consistency or construct validity. Comparing the results across different languages might lead to different response patterns. As a limitation of this study, we must admit that we had a relatively small sample size, which was further skewed due to the non-proportional distribution of the two surveyed professions. The sample size of this study may not appear to be particularly large at first glance, but in reality, the response rate, especially concerning the first course and the prospective veterinary medical assistants, was very high. Out of a total of 21 trainees working at the University of Veterinary Medicine, 13 signed up for the course. Compared to the first course, more participants in the second course showed interest in the learning intervention, with more registrations, thereby highlighting the relevance and readiness for interprofessional learning. To further generalise our results, comparable studies at the other four German veterinary institutions would be helpful. Generating a more proportional sample size of the different professions seems to be the biggest challenge because of the huge difference in the numbers of apprentices and students of the professions at universities. Therefore, the acquisition of veterinarian professions outside of the universities would help to balance the proportion. However, when examining the results concerning the various professions, it is important to note that even small changes in absolute numbers can reflect significant percentage changes. Also, the fact that we had various training occupations, such as biology laboratory technicians who have typically less patient and interprofessional contact as they often work in separate labs, may have influenced the needs assessment. The small sample size also leads to rare statistical findings and some tests simply are not possible with the low number of datasets. Nevertheless, the preliminary results suggest that we could have more significant findings with a larger sample size. This study also only rates the subjective perspective of the participants without having an objective measurement tool to compare with. The duration of the course (October 2022 to February 2023 and April 2023 to August 2023) may not have been sufficient for a significant change in attitudes, so a study comparing attitudes before and after the start of a degree course programme or training and after completion may yield more concrete results.

In both courses, 96.67% of the participants were female. This is slightly higher than the percentage reported by the Federal Chamber of Veterinary Surgeons (Bundestierärztekammer) concerning the student statistics of recent years [32]. These statistics show that the number of female veterinarians has increased significantly since 2002. In general, it has already been proven that women are more willing to participate in surveys [33], which can also be seen in other surveys conducted for veterinary students and veterinarians in Germany [23,34], and that female medicine and nursing students have a more positive attitude towards teamwork than male students [35,36]. The fact, that mostly females participated in our study could have had an impact on the results; referring to the mentioned studies, this impact seems to be a positive one. Also, the amount of datasets could be higher with a majority of female participants than with more male ones.

Based on our study, using the Readiness for Interprofessional Learning Scale, it becomes evident that the readiness for interprofessional learning is high. Following the initial course iteration, additional professions (e.g., animal keepers) reached out to us, expressing interest in the event. Implementing interprofessional interactions during education and training would be welcomed in further projects and curriculum adjustments and should not be limited to a few professional groups.

In conclusion, the preliminary evaluations indicate a high willingness towards interprofessional learning. The possibility of a shared communication course was well received in both university terms and was successfully completed on a voluntary basis. Regarding attitude change, initial indications are evident, which would most probably become more conclusive with a larger sample. These findings underscore the need for interprofessional learning during studies and training, as well as the desire and necessity to acquire communication skills. The results can be used to establish additional courses and programmes in this area.

## Figures and Tables

**Table 1:** Median of the RIPLS item answers of the paired samples of both courses (n = 60).

Subscale	RIPL Item	Pretest	Post-Test
Teamwork and collaboration	Learning with students will help me become a more effective member of a health care team.	3.28	3.93
	2. Patients would ultimately benefit if health care students worked together to solve patient problems.	4.70	4.55
	3. Shared learning with other health care students will increase my ability to understand clinical problems.	4.05	3.83
	4. Learning with health care students before qualification would improve relationships after qualification.	3.83	4.17
	5. Communication skills should be learned with other health care students.	3.87	4.03
	6. Shared learning will help me to think positively about other professionals.	3.75	4.05
	7. For small group learning to work, students need to trust and respect each other.	4.55	4.52
	8. Teamwork skills are essential for all health care students to learn.	4.43	4.40
	9. Shared learning will help me to understand my own limitations.	3.62	3.80
Professional identity	10. I do not want to waste my time learning with other health care students.	1.72	1.68
	11. It is not necessary for undergraduate health care students to learn together.	2.10	2.28
	12. Clinical problem-solving skills can only be learned with students from my own department.	2.33	2.25
	13. Shared learning with other health care students will help me to communicate better with patients and other professionals.	4.13	4.02
	14. I would welcome the opportunity to work on small-group projects with other health care students.	3.83	3.87
	15. Shared learning will help to clarify the nature of patient problems.	3.92	3.82
	16. Shared learning before qualification will help me become a better team worker.	3.83	4.07
Roles and responsibilities	17. The function of nurses and therapists is mainly to provide support for doctors.	3.07	3.13
	18. I am not sure what my professional role will be.	2.38	2.20
	19. I have to acquire much more knowledge and skills than other health care students.	3.22	3.02

**Table 2:** Demographic data of the paired samples from both courses (n = 60).

	Course 1		Course 2		Total
	Vet Students and Veterinarians	Vet Nurses, Laboratory Assistants, etc.	Vet Students and Veterinarians	Vet Nurses, Laboratory Assistants, etc.	
Female	15	6	30	7	58
Male	1	0	1	0	2
Previous communication training	7	1	7	1	16
Teamwork experience at work	16	5	29	6	59
Teamwork experience in free time	14	6	28	6	54

## Data Availability

All data presented in this study are available on request from the corresponding author. The data are not publicly available due to ethical and privacy restrictions.

## Supplementary Materials

The following supporting information can be downloaded at: https://www.mdpi.com/article/10.3390/ani14020229/s1, Table S1: Chi-square and PaBaK tests of the total pretests and post-tests and specifically for the professions; Table S2: *p*-values of the exact fisher test showing the difference of the answers between apprentices and students (paired samples: n = 60).
