# Supplementary material for: Measuring Veterinarian Professions’ Readiness for Interprofessional Learning in a Pre- and Post-Intervention Study
Source: Animals (Basel). 2024 Jan 11;14(2):229. doi: 10.3390/ani14020229 (PMC10812513; doi:10.3390/ani14020229)
Supplement: Supplementary file 1 [file animals-14-00229-s001.zip › animals-2776351-supplementary.pdf]

Supplementary table S1: Chi-square and PaBaK tests of the total pretests and posttests and specifically for the professions.

	Total			Students			Apprentices		
Item	McNemar	PaBaK	n=	McNemar	PaBaK	n=	McNemar	PaBaK	n=
1	0.9956	0.5127	59	0,9892	0.4837	46	0.5724	0.5385	13
2	0.6466	0.5111	60	0.6317	0.5461	47	1.0000	0.0769	13
3	0.5321	0.4915	59	0.8477	0,538	46	0.9473	0.3269	13
4	0.3208	0.3446	59	0.3329	0.3333	46	0.7660	0.3846	13
5	0.5966	0.3556	60	0.4485	0.3191	47	0.9536	0.4231	13
6	0.1727	0.2994	59	0.2328	0.1884	46	0.8088	0.6923	13
7	0.9037	0.3778	60	0.9004	0.3191	47	1.0000	0.3846	13
8	0.4460	0.4889	60	0.4012	0.6481	47	0.3173	0.3846	13
9	0.5066	0.2797	59	0.5268	0.2754	46	0.8318	0.1346	13
10	0.6340	0.4000	60	-	-	-	0.9698	0.5897	13
11	0.3882	0.2444	60	0.4745	0.2340	47	0.3208	0.2821	13
12	0.8300	0.1667	60	0.9798	0.2021	47	0.6317	-0.0256	13
13	0.8571	0.3556	60	0.8685	0.3191	47	-	-	-
14	0.3787	0.3556	60	0.6202	0.4043	47	0.5438	0.1795	13
15	0.3958	0.3778	60	0.3594	0.4043	47	0.6444	0.1923	13
16	0.4146	0.2542	59	0.7739	0.2624	47	0.7037	0.2222	12
17	0.7622	0.3750	60	0.8192	0.3883	47	-	-	-
18	0.6549	0.3750	56	0.7626	0.5021	44	0.6767	0.5556	12
19	0.8002	0.2105	57	0.9165	0.1193	44	0.9197	0.4872	13

Supplementary table S2: p-values of the exact fisher test showing the differences of the answers between apprentices and students (paired samples n= 60).

Question	Pretest 2022	Posttest 2022	Pretest 2023	Posttest 2023	Pretests total	Posttests total
1	0.8083	0.5257	1.0000	0.6680	1.0000	0.6197
2	0.3341	0.7658	0.6482	0.7890	1.0000	1.0000
3	0.5598	0.5700	0.8957	0.7927	0.4672	0.7498
4	0.7045	1.0000	0.6868	0.6306	0.8131	0.8187
5	1.0000	0.6217	0.7795	0.6893	0.8920	0.9033
6	**0.0098**	0.5872	0.0346	0.3020	0.0800	0.8594
7	0.6351	0.6364	0.7373	1.0000	1.0000	1.0000
8	1.0000	0.6524	1.0000	0.3063	0.7694	0.6476
9	0.6510	0.7087	0.1553	0.6786	0.0741	0.8558
10	0.3792	0.7346	0.2799	0.7737	0.1008	0.7781
11	1.0000	0.3680	0.7927	0.0923	0.9487	**0.0089**
12	0.2679	0.6172	0.1637	0.2206	0.2577	0.7170
13	0.3170	0,.316	0.8563	0.2428	0.9241	**0.0133**
14	0.5024	0.1914	1.0000	0.5015	0.6664	0.1527
15	0.2982	**0.0366**	1.0000	0.4676	0.8631	**0.0375**
16	0.6364	0.5356	0.1265	0.5593	0.1855	0.4333
17	0.2853	0.1679	0.7300	1.0000	0.1835	0.4550
18	0.3224	0.6679	0.6896	0.2861	0.5136	0.5362
19	0.4109	0.6960	0.7529	0.1195	0.4574	0.1730
